# Contribution of extracellular vesicles to neuroinflammation and cognitive and motor deficits in hyperammonemia and hepatic encephalopathy

**DOI:** 10.20517/evcna.2023.66

**Published:** 2024-01-17

**Authors:** Paula Izquierdo-Altarejos, Vicente Felipo

**Affiliations:** Laboratory of Neurobiology, Príncipe Felipe Research Centre, Valencia 46012, Spain.

**Keywords:** Hepatic encephalopathy, extracellular vesicles, motor incoordination, memory impairments, hippocampus, cerebellum, TNFα

## Abstract

Cirrhotic patients can present hepatic encephalopathy (HE), showing motor and cognitive deficits. Hyperammonemia and peripheral inflammation are known to induce neuroinflammation and alter neurotransmission, which finally induces neurological impairment in HE. However, the mechanisms by which the deleterious effects of peripheral inflammation are transmitted to the brain are not well understood. Extracellular vesicles (EVs) have recently emerged as a new mediator between the periphery and the brain, particularly in pathologies associated with sustained inflammation and in neurological disorders. In this work, we summarized the main findings on the role of plasma EVs in hyperammonemia and HE and discussed its potential implication in the pathogenesis of hepatic encephalopathy.

## HEPATIC ENCEPHALOPATHY

Acute or chronic liver failure can result in a complex neuropsychiatric disorder, hepatic encephalopathy (HE), which includes a wide spectrum of psychiatric and/or neurological disturbances, ranging from subclinical alterations to coma and even death^[1,2]^. Around 30% to 50% of patients with liver cirrhosis can develop minimal hepatic encephalopathy (MHE), which is clinically undiscernible and is characterized by alterations in cognition, psychometric speed, and executive functions, which result in reduced quality of life and shortened lifespan^[3]^.

### Pathogenesis

Hyperammonemia (HA) and peripheral inflammation act synergistically to induce neurological alterations in patients suffering from chronic liver disease^[4,5]^. Liver damage induces both hyperammonemia and peripheral inflammation, leading to neuroinflammation, altering neurotransmission, and ultimately inducing the cognitive and motor impairment characteristic of hepatic encephalopathy^[1,2,6]^. However, the mechanisms by which peripheral inflammation induces neuroinflammation are not completely understood and are difficult to study in patients. Mechanistic studies have been performed in animal models, including surgical models such as rats with portacaval shunts and non-surgery models such as rats with chronic HA, which is an established model for HE and reproduces the motor and cognitive impairment found in patients with MHE (reviewed by Cabrera-Pastor *et al.*^[7]^). Some mechanisms that have been suggested are the activation of cytokine receptors in endothelial cells by pro-inflammatory cytokines^[8]^ or the infiltration of peripheral immune system cells such as lymphocytes and monocytes into the brain^[9,10]^.

## EXTRACELLULAR VESICLES AS NOVEL MEDIATORS BETWEEN THE PERIPHERY AND THE BRAIN

More recently, extracellular vesicles (EVs) have been suggested as an alternative mechanism for communication between the periphery and the brain in HE^[2,11-13]^. The main reasons to study the contribution of EVs in the induction of neuroinflammation and the subsequent cognitive and motor impairment in HE are their ability to trespass the blood-brain-barrier (BBB) and the results reported in previous studies pointing out their relevance as intercellular mediators in different pathologies associated with chronic inflammation, including neurological diseases.

Several studies have demonstrated that EVs in peripheral blood can reach the brain and exert a functional effect. Moreover, this process seems to be facilitated in inflammatory conditions. For example, Ridder *et al.* designed an experiment with transgenic mice that only expressed the Cre recombinase in cells of the hematopoietic lineage^[14]^. Then, they isolated the EVs from the serum of these mice and injected them into control mice, observing that there was a functional transfer of RNAs from the injected EVs to different neuronal types in the brain, especially to Purkinje neurons, as these cells acquired the ability to express the Cre recombinase. This process was favored by peripheral inflammation and was sufficient to induce relevant changes in the recipient cells, altering the expression profile of miRNAs in Purkinje neurons that had taken up RNA from the injected EVs. Li *et al.* demonstrated that fluorescently labeled EVs from the serum of mice with LPS-induced endotoxemia reached the hippocampus 24 h after intravenous injection into control mice, inducing microglia activation and astrogliosis in hippocampus, as well as increased levels of TNFα and IL-6^[15]^. Matsumoto *et al.* found that erythrocytes from patients with Parkinson’s disease release α-synuclein-enriched EVs, which can cross the BBB and increase the inflammatory response in microglia^[16]^. A different study also shows that EVs isolated from patients with Parkinson’s disease were able to induce similar alterations to those found in patients when injected into control mice, such as dopaminergic neuron degeneration and microglia activation, as well as motor symptoms such as movement alterations^[17]^. EVs could also have a role in Alzheimer’s disease pathogenesis, since EVs in the CSF contain high levels of Aβ, which is toxic to neurons cultured *in vitro*^[18]^ and in animal models *in vivo*^[19]^, and Tau protein, which can be transmitted to neurons, inducing Tau aggregation^[20,21]^.

### Evidence of the contribution of EVs to motor and cognitive impairment in animal models of hepatic encephalopathy

**Figure 1:**
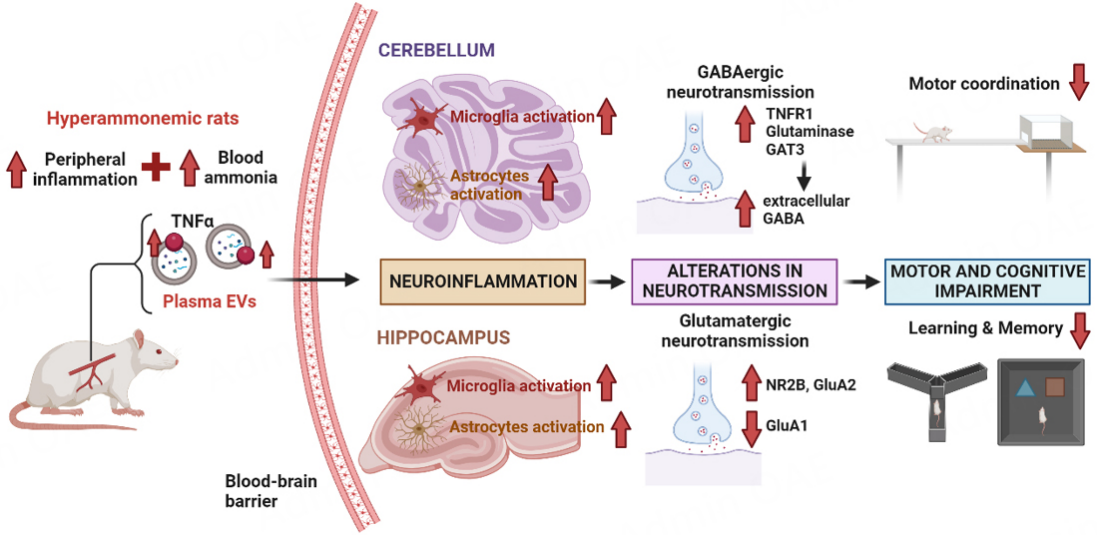
Summary of the main effects of plasma EVs from hyperammonemic rats on cerebellum and hippocampus.

Recent studies carried out in animal models demonstrate that peripheral EVs isolated from the plasma of hyperammonemic rats (HA-EVs) can reach the brain and induce motor and cognitive deficits when injected into control rats. The effects on two brain areas have been studied so far: cerebellum, mainly related to motor alterations; and hippocampus, mainly related to cognitive impairment. The main findings observed to date are summarized in Figure 1.

Izquierdo-Altarejos *et al.* demonstrated for the first time that intravenously injected HA-EVs reached the cerebellum of control rats, co-localizing with Purkinje neurons and microglia^[11]^. HA-EVs induced neuroinflammation in cerebellum, with microglia and astrocyte activation, and lead to motor incoordination in control rats. The mechanism leading to motor alterations was further studied by Izquierdo-Altarejos *et al.*^[12]^. In this work, the authors used an *ex vivo* approach to better understand the molecular mechanisms involved. They showed that *ex vivo* incubation of cerebellar slices from control rats with HA-EVs reproduced the microglia and the astrocytes activation found *in vivo* and enhanced GABAergic neurotransmission by two main mechanisms: increasing the TNFR1-NF-kB-glutaminase-GAT3 pathway and the TNFR1-CCL2-BDNF-TrkB-KCC2 pathway, which would be responsible for the motor incoordination observed *in vivo*. HA-EVs would trigger the activation of TNFR1 in cerebellum, inducing the nuclear translocation and activation of NF-κB in Purkinje neurons, which in turn increases the content of glutaminase I and the content and membrane expression of the GABA transporter GAT3. Cabrera-Pastor *et al.* found that an increase in glutaminase I in the cerebellum of HA rats leads to higher levels of extracellular glutamate, which is taken up along with Na^+^ by activated astrocytes^[22]^. This alteration in the sodium gradient reverses the function of GAT3, leading to the release of GABA to the extracellular space, enhancing GABAergic neurotransmission. More recently, it has been observed that the activation of TNFR1 in the cerebellum of hyperammonemic rats can also contribute to enhancing GABAergic neurotransmission by an alternative mechanism: TNFR1 would increase the CCL2 production in Purkinje neurons, which interacts with its receptor CCR2 in activated microglia, resulting in higher production of BDNF, as reported by Arenas *et al.*^[23]^. This results in an increase in and activation of the BDNF receptor TrkB and the chloride co-transporter KCC2, especially in Purkinje neurons, which reduces the intracellular Cl^-^ concentration and enhances the activation of GABA_A_ receptors. HA-EVs induced similar effects to that found in HA rats when added to cerebellar slices *ex vivo*, suggesting that the activation of these two pathways would explain the induction of motor deficits *in vivo*^[12]^.

HA-EVs have also been found to induce neuroinflammation in hippocampus, altering neurotransmission and eliciting cognitive impairment in control rats, which further supports their contribution to neurological alterations in HE^[13]^. In this work, authors observed that HA-EVs also reached the hippocampus when injected into control rats, inducing microglia and astrocyte activation and increasing the levels of the pro-inflammatory cytokines IL-1β and TNFα, both *in vivo* and *ex vivo*. The injection of HA-EVs induced cognitive alterations, impairing memory and learning in control rats, exhibiting reduced short-term memory, object location, and object recognition memory, reproducing the deficits observed in HA rats. In hyperammonemic rats, it is known that these deficits are a consequence of increased IL-1β levels in hippocampus, which affect glutamatergic neurotransmission, altering the membrane expression of AMPA (GluA1 and GluA2 subunits) and NMDA (NR2B subunit) receptors^[24]^. The *ex vivo* study of the HA-EVs effects on the hippocampus showed that they can induce cognitive impairment by similar mechanisms. Hippocampal slices from control rats incubated *ex vivo* with HA-EVs showed NF-κB activation both in neurons and microglia, which would increase the IL-1β production. IL-1β would interact with its receptor IL-1R and modulate the membrane expression of NMDA receptors, increasing the membrane expression of the NR2B subunit. This would result in the alterations found in GluA1 and GluA2 subunits of AMPA receptors. Altogether, these alterations in glutamatergic neurotransmission would be responsible for memory impairments^[13]^.

Another interesting finding of these studies is the increase in the number of circulating EVs found in the plasma of HA rats^[11,13]^. Although the mechanism has not been described in detail, several studies report an increase in the EVs production in different pathologies such as alcoholic hepatitis^[25]^, β-thalassemia^[26]^, obesity and diabetes miellitus^[27]^, chronic obstructive pulmonary disease^[28]^ and autism spectrum disorders^[29]^.

It is noteworthy that the injection or the *ex vivo* addition of EVs from control rats did not induce significant pathological effects in the studies presented above^[11-13]^. This indicates that hyperammonemia not only increases EVs in plasma, but also changes its composition, as their effects are significantly different from that of EVs from control rats. Izquierdo-Altarejos *et al.* studied the protein cargo of EVs from control and HA rats, reporting differences in proteins associated with biological processes of response to stimulus, immune system, glutamine/glutamate conversion, metabolic processes, integrin, and FAS signaling pathways^[11]^. They also found significantly higher levels of TNFα in HA-EVs, whose role will be further discussed in the following section.

### Relevance of the EVs TNFα in the transmission of peripheral inflammation to the brain and the impairment of neurological function

As mentioned, TNFα, as well as its receptor TNFR1, is increased in EVs from the plasma of hyperammonemic rats^[11]^. Some studies in the literature suggest a role for the TNFα present in EVs in the induction of inflammatory effects in certain pathologies. Zhang *et al.* observed that EVs isolated from synovial fibroblasts of patients with rheumatoid arthritis carried a transmembrane form of TNFα, which was able to activate the NF-κB pathway and induce MMP-1 expression in fibroblast cultures, and to promote proliferation and resistance to apoptosis when added to CD4+ T lymphocyte cultures, suggesting that TNFα in the EVs could be contributing to joint inflammation^[30]^. Obregon *et al.* observed that LPS-activated dendritic cells secreted vesicles carrying both soluble TNFα and its receptors TNFR1 and TNFRII^[31]^. These EVs were internalized by epithelial cells and induced the release of pro-inflammatory molecules such as IL-8, CCL2, CCL5, MIP-1β, or G-CSF by activating the transcription factor NF-κB. Gao *et al.* reported that mature dendritic cells derived EVs increased endothelial inflammation and atherosclerosis, increasing inflammation and adhesion markers, via membrane TNFα both *in vivo* and *in vitro*, and also mediated by NF-κB pathway activation^[32]^.

TNFα would be one of the main triggers of the pathways leading to alterations in the neurotransmission that lead to motor and cognitive impairment in hyperammonemia and hepatic encephalopathy. Blocking TNFα in the surface of the HA-EVs by incubation with infliximab (an antibody against TNFα) prevented microglia and astrocyte activation and the activation of the TNFR1-NF-κB-glutaminase-GAT3 and the TNFR1-CCL2-BDNF-TrkB-KCC2 pathways in cerebellum *ex vivo*^[12]^. Similarly, HA-EVs did not induce microglia and astrocyte activation in hippocampal slices of control rats when they were previously incubated with infliximab. Blocking TNFα also prevented the alterations in AMPA and NMDA receptors associated with cognitive deficits in hyperammonemic rats^[13]^.

Infliximab is currently being used as a pharmacological treatment in patients with rheumatoid arthritis and Chron’s disease, with the aim of reducing peripheral inflammation^[33,34]^. In hyperammonemic rats, infliximab treatment was effective in preventing the induction of peripheral inflammation and the subsequent alterations in hippocampus, including microglia and astrocyte activation, the increase in IL-1β and TNFα and the alterations in membrane expression of glutamatergic receptors that finally lead to spatial memory impairment^[35]^. Interestingly, Izquierdo-Altarejos *et al.* observed that peripheral treatment with infliximab also normalized the number of EVs in the plasma of hyperammonemic rats and their TNFα levels^[13]^. These EVs were no longer inducing neuroinflammation *ex vivo* when added to hippocampal slices of control rats, which supports that TNFα in the EVs is one of the key factors for the transmission of pathological processes to the brain^[13]^. These results also suggest that peripheral inflammation is responsible for the alterations in the cargo of circulating EVs in hyperammonemic rats.

### Alterations in peripheral EVs found in patients with hepatic encephalopathy

The study by Gallego *et al.* was the first to characterize circulating EVs in patients with MHE^[36]^. The authors compared the miRNA and protein cargo of plasma EVs isolated from cirrhotic patients (with and without MHE) and control volunteers: EVs from MHE patients were enriched in inflammatory factors, such as TNFα, ADAM17 or miR-130b-5p and in some cellular markers (L1CAM, neuronal marker; CD4, from T helper lymphocytes; CD8, from T cytotoxic lymphocytes; and CD19, from B lymphocytes), which could indicate a dysregulation in these cell populations. They also studied the effects of EVs on CD4+ T lymphocyte cultures, finding that EVs isolated from cirrhotic patients with MHE modulated the expression of anti-inflammatory (TGFβ) and pro-inflammatory (IL-17, IL-21, and TNFα) cytokines *in vitro*. These alterations found in the lymphocytes were similar to those observed in the immunophenotype of MHE patients in a previous study^[37]^. The results obtained by Gallego *et al.* show that EVs could play an important role in the induction of immune changes observed in MHE patients^[36]^.

## CONCLUSIONS

EVs emerge as an additional mechanism that contributes to transmitting peripheral inflammation to the brain in hyperammonemic rats, inducing neuroinflammation and impairing motor and cognitive function. A deep understanding of the mechanisms involved in this process opens the possibility of targeting EVs and their altered cargo in order to design new treatments that would improve motor and cognitive function in patients with hepatic encephalopathy.

## DECLARATIONS

### Copyright

© The Author(s) 2024.
